# Tracking changes in physical activity during inpatient treatment in a psychiatric clinic in Germany by asking two simple questions

**DOI:** 10.1007/s00406-023-01565-2

**Published:** 2023-02-11

**Authors:** Jannik Roempler, Moritz Bruno Petzold, Antonia Bendau, Jens Plag, Andreas Ströhle

**Affiliations:** 1 Department of Psychiatry and Neurosciences, CCM, Charité–Universitätsmedizin Berlin, Corporate Member of Freie Universität Berlin and Humboldt Universität zu Berlin, Charitéplatz 1, 10117 Berlin, Germany; 2 HMU Health and Medical University Potsdam, Potsdam, Germany; 3 Oberberg Fachklinik Potsdam, Potsdam, Germany

**Keywords:** Exercise, Training, Sport, Psychiatry, Vital sign, Mental health

## Abstract

Increasing physical activity is essential to improve psychiatric patients’ physical and mental health. This study aimed to characterise the physical activity levels of inpatients in a general psychiatric clinic and to determine the feasibility of using a simple tool in everyday practice to assess physical activity levels in standard patient documentation. We assessed the level of physical activity undertaken by patients treated on an inpatient basis in a psychiatric hospital over 20 months. A total of 328 patients were included in the analysis. Physical activity was measured using a slightly altered version of the Exercise as a vital sign (EVS) questionnaire. All information was extracted from letters of discharge. During inpatient treatment, moderate to vigorous activity levels increased, and more patients engaged in physical activity. Patients with mood or anxiety disorders displayed the most considerable increase in physical activity. Patients with other diagnoses, such as schizophrenia, benefitted less or not at all. Factors associated with physical activity included—among others—history of substance use, education and month of admission. Investigating the feasibility of standardised documentation of physical activity showed fluctuation in documentation rates throughout the study. The level of physical activity performed by psychiatric patients can be increased during inpatient treatment. Implementing physical activity level as part of standard patient documentation is a first step in gathering data to assess the need for interventions to achieve an optimal physical activity in psychiatric patients throughout inpatient treatment.

**Supplementary Information:**

The online version contains supplementary material available at 10.1007/s00406-023-01565-2.

## Introduction

### Physical activity

Physical activity is beneficial for mental and physical health. It has been shown to decrease the incidence and prevalence of anxiety disorders and depression [1–6]. A reduction of symptoms through physical activity has been shown in many psychiatric disorders, such as anxiety disorders, depression, post-traumatic stress disorder, substance use disorders and schizophrenia [1, 3, 4, 7–9]. Of great importance is that physical activity has been shown to improve the quality of life of patients with mental disorders [8].

Physical activity also has a positive effect on a patient’s physical health. Physical activity is associated with a decrease in Body Mass Index (BMI) [10, 11], a lower incidence of diabetes, a lower incidence of cancer [12] and a reduction in blood pressure and cardiovascular events [12, 13]. In addition, the connection between physical health and mental health was shown by Kandola et al. whose study showed that patients with a lower cardiorespiratory fitness (CRF) [14] had a higher risk of mental disorders. Hence, improving physical health may also result in improving mental health.

Approximately 27.5% of the population worldwide is engaging in insufficient physical activity. This number is lower in southeast Asia and higher in high-income western countries (43.2%) [15, 16]. Another study found even higher inactivity rates [17]. Due to this “epidemic of physical inactivity”, 5.3 million deaths occur worldwide each year, leading to a decrease in global life expectancy by 0.68 years [12]. Furthermore, studies have shown that patients with mental disorders engage in significantly less physical activity than the general population [18–22]. Due to the vulnerable nature of this patient group, more effort must be made to increase physical activity.

Globally, 32.4% of years lived with disability are due to mental disorders and 13% of disability-adjusted life-years result from mental disorders [23]. In Germany, 27.8% of the population suffers from a mental disorder [24, 25]. These numbers highlight that mental disorders are a prevalent societal problem, meaning that increasing physical activity could benefit many subjects.

### Exercise is medicine

*Exercise is Medicine* is an initiative using evidence-based approaches to increase physical activity in medicine. It was introduced in 2007 by the *American Medical Association* and the *American College of Sports Medicine* [26]. *Exercise is Medicine* is present in more than 40 countries worldwide, with numbers still increasing [26]. The three steps in the *Exercise is Medicine* initiative include [26]:

1. Assessing the physical activity level of each patient
2. Counselling on physical activity
3. Referring to exercise and fitness professionals

Physical activity assessment is done by implementing exercise as a vital sign (EVS) [27], meaning that when routine vital signs are taken from patients as part of the documentation process, the level of physical activity is also obtained. Previous studies showed that implementing the EVS in primary care resulted in increased physical activity, decreased weight and decreased HbA1C (a marker for long-term blood sugar levels) [28, 29]. Even though EVS showed promising results in the primary care setting, to our knowledge, it has not yet been implemented in a psychiatric setting. Implementing this may be a cost-effective way to promote the positive effects of physical activity for patients with mental disorders.

### Aim of our study

Information on the physical activity levels of inpatients in psychiatric hospitals is scarce. However, it is necessary to identify interventions that should be introduced to optimise physical activity in patients with mental disorders. Therefore, this study aimed to implement physical activity as a vital sign in a psychiatric unit and, through this process, identify roadblocks along the way to allow other psychiatric units to do the same. The documentation rate is a way to track clinician adherence to implementing physical activity as a vital sign. Furthermore, this analysis aimed to identify whether physical activity changes during inpatient treatment. Also, factors were determined that might influence the amount of physical activity performed by patients.

The primary outcome was to find changes in physical activity levels during inpatient treatment. Based on these changes, we analysed patient compliance with WHO recommendations and other differences in activity levels achieved by different patient groups. These groups were sorted by psychiatric diagnosis and/or resident ward.

As secondary outcomes, we examined factors that might influence physical activity. By doing so, we wanted to explore whether there are subgroups that are especially in need of proactive work to increase physical activity levels and therefore help patients across all mental disorders to claim the benefits that physical activity has to offer.

## Methods

### Study design and study population

In this retrospective chart review of routine data, we included all patients of the Department of Psychiatry and Neurosciences of the Charité–Universitätsmedizin Berlin, Campus Mitte, from July 2019 to February 2021. An optionally closed ward (OCW1) primarily for patients with mood [affective] disorders (up to 17 patients), an open ward (OW) for general psychiatric patients (up to 20 patients), a day clinic ward (DCW) for all psychiatric disorders (up to 18 patients) and an optionally closed ward (OCW2) primarily for patients with schizophrenic disorders (up to 17 patients). In early 2020, the number of patients per ward was reduced due to the capacities needed for COVID-19 patients. Patients suffering from all mental disorders were eligible for inclusion in this study. Patients were excluded from this analysis if the physical activity was not documented correctly or other key data were missing.

This analysis was approved by the local ethics committee (EA1/293/20) and did not need to be registered as a study as it is a retrospective analysis of routine clinical data.

### Implementation

To implement physical activity as part of standard patient documentation, we introduced the proposed method to clinical physicians working on the abovementioned wards and the senior physicians in charge. After implementing this into standard documentation procedures, regular talks and written communication (at least once a month) with physicians were needed to continuously increase and maintain the rate of correctly assessed and documented physical activity levels. New physicians were briefed about the project and the documentation of physical activity by their senior physicians. In addition, every month, we evaluated the documentation rates of physical activity in the letters of discharge. If a physician did not document the physical activity, we contacted this physician to emphasise the importance of this project and explained the documentation process to guarantee future documentation.

### Measures

Following the idea of the *Exercise is Medicine* initiative, we implemented the following questions into our routine patient assessment at the beginning and end of inpatient treatment:

1. “On average, how many days per week do you engage in moderate to strenuous exercise (like a brisk walk)?” [27]
2. “On average, how many minutes do you engage in exercise at this level?” [27]

We slightly altered the questions used by the *Exercise is Medicine* initiative by omitting the word "exercise" and replacing it with “Physical activity”. Fortier et al. promoted this change to reach a broader group of people [30]. We also included a broader spectrum of physical activities, as the WHO classifies exercise and activities during work and leisure time as beneficial [31]. By asking these two questions on the day of admission and the day of discharge, we implemented an easy and time-efficient tool into our routine assessment to evaluate the physical activity of psychiatric patients at our clinic. The responsible clinician documented the results of these questions as minutes per week of moderate to strenuous physical activity in the discharge letter. This information was later retrieved from the discharge letter and used in our analysis. In addition, other possible factors that may influence the level of physical activity achieved by patients were also obtained from the discharge letter.

The factors obtained were: age, gender, BMI, education, housing situation, the status of employment, principal diagnosis, additional diagnoses, ward, legal guardianship, residence status, the month of admission, year of admission, duration of treatment, alcohol consumption, nicotine consumption, drug consumption and medication. Psychiatric diagnoses were procured from patient medical records based on ICD-10 criteria used by a board-certified psychiatrist. Additional somatic diagnoses were obtained from patient medical records based on ICD-10 criteria used by board-certified physicians.

The change in physical activity, the compliance with the WHO recommendation (150 min of moderate to vigorous physical activity per week) and the effect of resident wards and psychiatric diagnosis on physical activity were defined as primary outcomes. All other variables were measured as secondary outcomes to find factors influencing physical activity. If physicians were unable to assess patients' physical activity due to various reasons (such as a delusional state of the patient), physical activity was documented as “not assessable”. The documentation rate of physical activity was used to determine the success of implementing physical activity as a vital sign and especially the adherence of clinicians involved.

### Physical activity recommendation

According to WHO guidelines, a person is sufficiently physically active when accumulating either 150–300 min of moderate or 75–150 min of vigorous physical activity (or a combination) per week [31–33]. To keep physical activity assessment as simple as possible, we used the simpler version of the WHO recommendation of 150 min of moderate to vigorous physical activity per week (as is used in *Exercise is Medicine*) [34]. This recommendation has been used in previous publications as well.

### Statistical analysis

We used IBM SPSS 25.0 to conduct the statistical analysis. Where data were not normally distributed, non-parametric tests were applied. We conducted the Wilcoxon signed rank test to test for significance in the change of physical activity and compliance with the WHO recommendations of 150 min of moderate to vigorous physical activity per week. We used the Kruskal–Wallis test to discover significant differences in physical activity between principal diagnosis and/or resident ward groups. The Kruskal–Wallis test was also used to examine further factors significantly affecting physical activity levels. These included BMI, education, housing situation, the status of employment, month of admission, year of admission, alcohol use, nicotine use, drug use, depression severity and age categories. For gender, legal guardianship, and residence status we used the Mann–Whitney *U* test. Finally, we used Spearman’s rank correlation test to search for correlations between the level of physical activity and the duration of treatment. For all tests, the significance level was set to *p* = 0.05 using two-tailed tests.

## Results

### Study population

Out of the 1051 patients discharged from July 2019 to February 2021, the physical activity was not documented or documented incorrectly in 604 (57%) patients. In 119 out of the 447 correctly documented patients, the physical activity was not assessable. As a result, 328 out of 1051 (31.2%) patients were included in this analysis. A demographic overview of the study population can be found in Table 1.

**Table 1:** Demographic overview of the study population

		*n*	%
Gender	Male	176	53,7%
	Female	152	46,3%
	Non-binary	0	0,0%
Age categories	18–30	67	20,4%
	31–40	82	25,0%
	41–50	78	23,8%
	51–64	74	22,6%
	> 64	27	8,2%
BMI categories	Underweight	3	3,5%
	Normal weight	42	49,4%
	Overweight	23	27,1%
	Adipose	17	20,0%
Level of education	No qualification	18	7,1%
	Secondary modern school qualification	8	3,1%
	High-school diploma	17	6,7%
	Higher School Certificate	25	9,8%
	Completed vocational training	84	32,9%
	With a degree	103	40,4%
F0-F9	F0	6	1,8%
	F1	63	19,2%
	F2	86	26,2%
	F3	140	42,7%
	F4	18	5,5%
	F5	1	0,3%
	F6	11	3,4%
	F7	0	0,0%
	F8	1	0,3%
	F9	2	0,6%

A demographic overview of the study population is displayed containing age, gender, BMI, level of education and the main psychiatric diagnosis. The values are given in absolute numbers and percentages

**Fig. 1:**
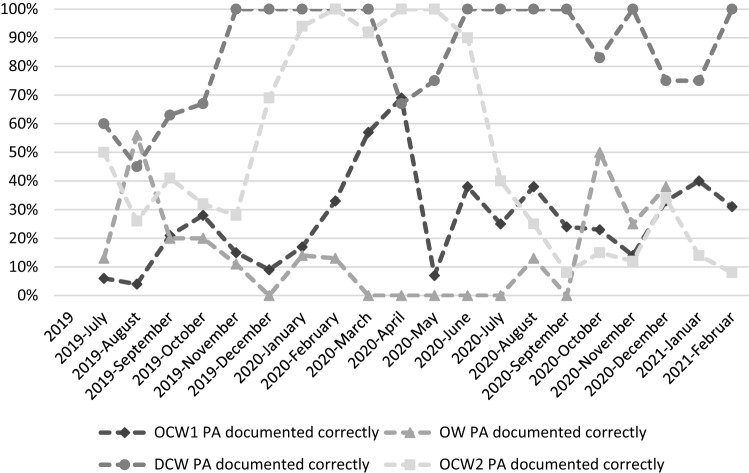
Documentation of Physical activity. The rates of correctly documenting physical activity on optionally closed ward 1 (OCW1), open ward (OW), day clinic ward (DCW) and optionally closed ward 2 (OCW2) are shown from July 2019 to February 2021. The documentation rate is displayed in percent

During the introduction of physical activity as a vital sign, the documentation rates of physical activity fluctuated (Fig. 1).

### Prevalence of physical activity and compliance with WHO recommendations

**Fig. 2:**
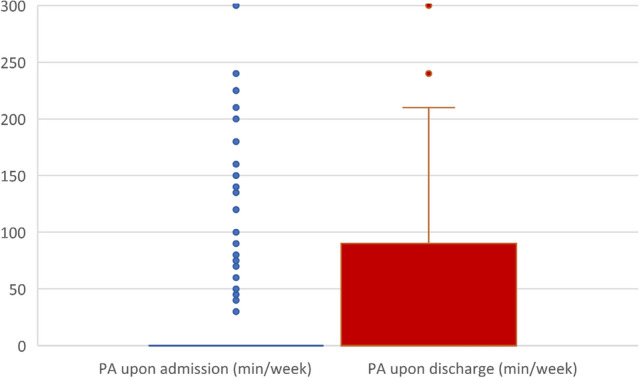
Change in physical activity during inpatient treatment. Note. The boxplots show moderate to vigorous physical activity (PA) in minutes per week (min/week) for admission and discharge

Information on physical activity was available for all 328 patients included in this study. The average minutes spent performing moderate to vigorous physical activity per week increased significantly by 19.04 min (SD = 58.81, Range: − 300 to 328; asymptotical Wilcoxon-Test: *z* = − 6.175, *p* = 0.000, *n* = 328), from *M* = 39.66 min (SD = 100.39, Range: 0–900) upon admission to *M* = 58.70 min (SD = 107.33, Range:0–900) upon discharge (Fig. 2).

**Fig. 3:**
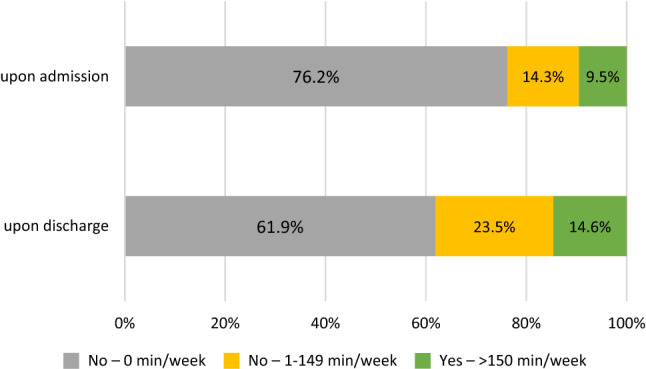
Compliance with WHO recommendations of 150 min of moderate to vigorous physical activity. Note. Compliance with the WHO recommendations of 150 min of moderate to vigorous physical activity is shown upon admission and discharge. The results are given in percent and distributed into 3 categories: No-0 min/week (min/week), No-1-149 min/week (min/week), Yes- > 150 min/week (min/week)

Upon admission, only a small fraction of patients met the WHO recommendation of 150 min of moderate to vigorous physical activity per week, and more than three-quarters of patients were not engaging in any physical activity. Figure 3 shows that the fraction of physically inactive patients decreased by 14.3% from admission to discharge. In contrast, the fraction of patients engaging in an insufficient amount of physical activity and the fraction of patients engaging in a sufficient amount of physical activity increased significantly from 14.3 to 23.5% and 9.5 to 14.6% (asymptotical Wilcoxon-Test: *z* = − 6.342, *p* = 0.000, *n* = 328), respectively.

### Physical activity in different mental disorders

**Fig. 4:**
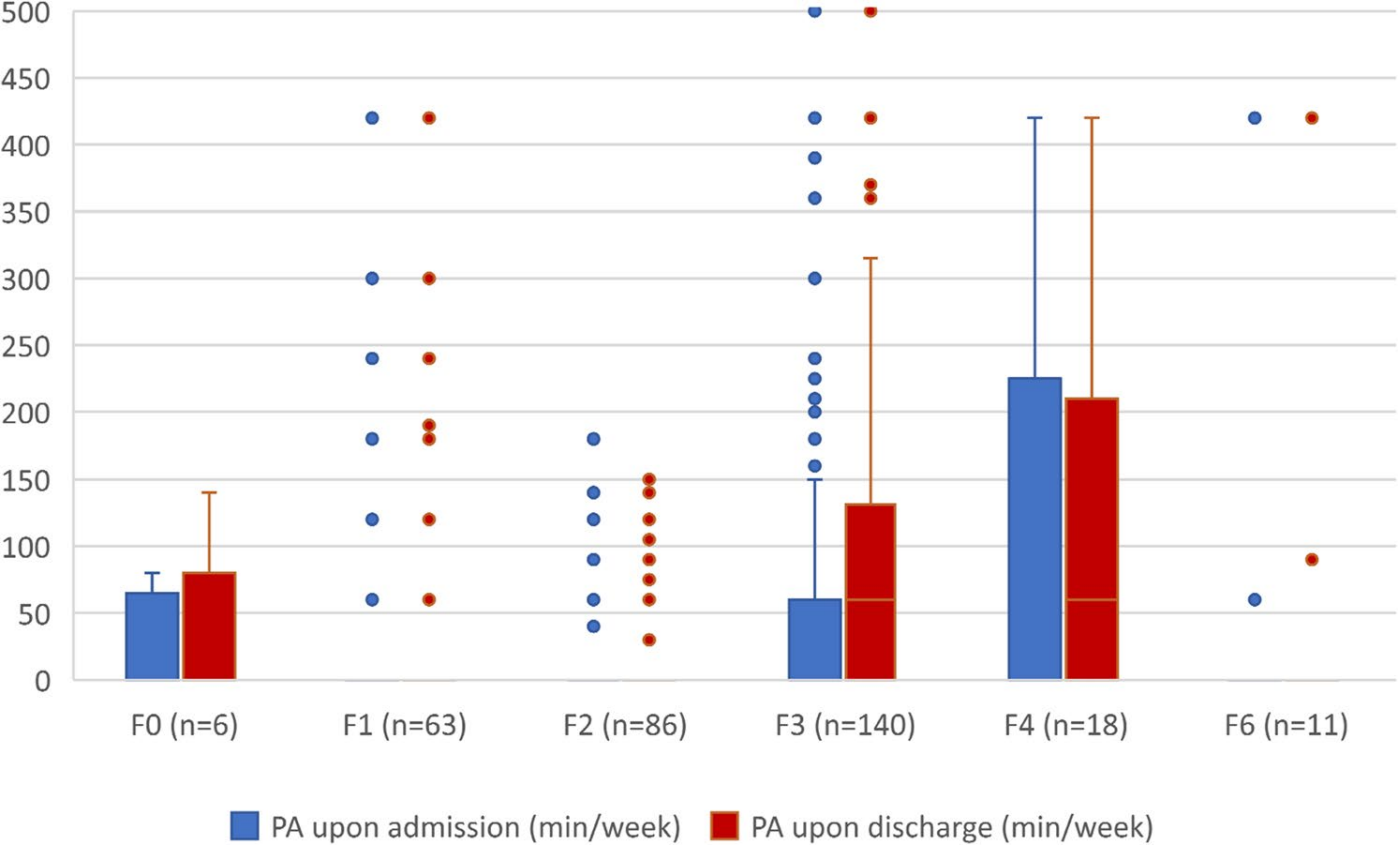
Physical activity in different mental disorders. Note. The boxplots show moderate to vigorous physical activity (PA) in minutes per week (min/week) for different mental disorders upon admission and upon discharge. F0 = organic, including symptomatic, mental disorders; F1 = mental and behavioral disorders due to psychoactive substance abuse; F2 = schizophrenia, schizotypal, and delusional disorders; F3 = mood disorders, depression, and bipolar disorders; F4 = neurotic, anxiety, stress-related, and somatoform disorders; F6 = disorders of adult personality and behaviors; F9 = behavioral and emotional disorders with onset usually occurring in childhood and adolescence

Figure 4 shows the difference in physical activity achieved by patient groups with different mental disorders. On one end of the spectrum, patients with neurotic, anxiety, stress-related, and somatoform disorders showed the highest level of physical activity with an average of 111.11 min (SD = 170.15) of moderate to vigorous physical activity per week. On the other hand, patients with schizophrenic, schizotypal, or delusional disorders showed, on average, the lowest level of physical activity, with *M* = 10.12 min (SD = 33.00).

However, the numerically most considerable increase in physical activity was seen in patients with a mood (affective) disorder diagnosis, with an increase of 35.54 min (SD = 77.73) of moderate to vigorous physical activity per week between admittance and discharge. Notably, patients with neurotic, anxiety, stress-related, or somatoform disorders also increased their physical activity by 18.89 min (SD = 48.86).

Upon discharge, patients with schizophrenic, schizotypal, or delusional disorders remained with the lowest physical activity. In contrast, patients with neurotic, anxiety, stress-related, or somatoform disorders, as well as patients with a mood (affective) disorder diagnosis, showed the highest physical activity with an average of 130.00 (SD = 157.11) and 87.68 min (SD = 123.72) of moderate to vigorous physical activity per week, respectively. Further details can be found in Table S1 of the supplements.

### Physical activity on different wards

We also compared physical activity between the different wards. Upon admission, the open ward (OW) showed the highest physical activity, averaging 147.78 min (SD = 165.09) of moderate to intense physical activity per week. Conversely, patients on the two optionally closed wards (OCW) reported the lowest physical activity per week, with an average of 39.61 (SD = 91.43; OCW1) and 17.82 min (SD = 57.74; OCW2), respectively.

During inpatient treatment, patients on the day-clinic ward reported the biggest increase in physical activity per week between admission and discharge, with 51.67 min (SD = 74.80). Despite the high physical activity upon admission, the increase of physical activity at discharge amongst patients of the open ward was only *M* = 16.67 min per week (SD = 86.84). However, on optionally closed ward1 (OCW1), patients increased their physical activity by only 11.78 min per week (SD = 52.30). Patients on the second optionally closed ward (OCW2) actually decreased in average physical activity between admission and discharge by 0.60 min per week (SD = 24.38). Further details can be found in table S2 in the supplements.

### Analysis of secondary outcomes

Our secondary outcomes were based on an exploratory analysis of factors that might be associated with the levels of physical activity. In the exploratory analysis (see results in supplement Table S3), we used Mann–Whitney *U*, Kruskal Wallis, and Spearman’s rank correlation tests. We found that education, housing situation, employment status, legal guardianship status, residence status, month of admission, year of admission, alcohol intake, nicotine use, drug use, depression severity and duration of treatment were associated with physical activity.

Patients with higher education showed higher physical activity upon admission and a higher increase in physical activity throughout inpatient treatment. Living in a private home showed higher physical activity levels upon admission and discharge compared to living in therapeutical housing or without a fixed abode. Employed patients showed higher physical activity levels upon admission and discharge compared to unemployed patients or patients still in education. A legal guardianship was associated with lower physical activity levels. In cases of non-voluntary residency in the psychiatric clinic, lower physical activity levels were reported, compared to voluntary residency. If patients were admitted during May, July, or August, higher physical activity levels were registered upon admission and discharge. In 2020, patients displayed lower physical activity levels upon admission, compared to 2019 and 2021. Patients with an irregular alcohol, nicotine or drug use reported higher physical activity than patients with no consumption, regular consumption, or substance abuse. Patients with moderate or severe depression showed a higher increase in physical activity levels during inpatient treatment, compared to patients with mild depression or no depression. Finally, the longer the duration of the inpatient treatment, the higher the increase in physical activity.

On the other hand, age, gender, BMI and comorbidities showed no association with the levels of physical activity.

## Discussion

We found that during inpatient treatment, moderate to vigorous physical activity levels increased, leading to a greater number of patients engaged in physical activity and, therefore, more patients complying with the 150 min recommendation given by the WHO. The activity levels of patients with a neurotic, anxiety, stress-related, and somatoform disorder diagnosis and of patients with a mood (affective) disorder diagnosis increased the most. In contrast, the activity level of patients with other psychiatric diagnoses changed less or not at all. Furthermore, patients engaged in different amounts of physical activity depending on the kind of ward they resided on. We also found further factors that may influence the level of physical activity.

### Documentation of physical activity

As shown in Fig. 1, the documentation rate increased until around July 2020. However, after July 2020, a decrease in the documentation rate was observed. One reason for this might have been the ongoing COVID-19 pandemic, as some psychiatric wards reduced their capacity, and less attention was granted to the documentation of physical activity. Whether the COVID-19 pandemic influenced physical activity is challenging to analyse in this study. Lower documentation rates and patient numbers do not allow a direct comparison of physical activity pre- and post-pandemic. Due to these circumstances, this limitation may heavily influence the comparison of 2019, 2020 and 2021.

### Physical activity levels

We found that during inpatient treatment, the average (moderate to vigorous) physical activity per week increased significantly by 19.04 min. Upon admission, 39.7 min increased to 58.7 min upon discharge. Previous studies found higher physical activity in inpatients and outpatients. For example, in a meta-analysis by Vancampfort et al. a mean physical activity of 90.1 min per day was found in psychiatric inpatients [35]. However, the analysis includes studies with a broad spectrum of mental disorders and physical activity levels, of which some display similar physical activity levels to our study. Furthermore, a considerable variation of programs implemented to promote physical activity in featured clinics can be seen [36]. This feature would lead to a larger dispersion in physical activity levels recorded.

Petzold et al. found an average of 288 min per week of self-reported physical activity in psychiatric outpatients [22]. Chapman et al. found similar results, with 270 min per week of self-reported physical activity in psychiatric outpatients [37]. Compared to our study, the higher physical activity seen in these studies may result from different factors. One, physical activity levels differ between outpatient and inpatients. Studies have shown higher physical activity among outpatients [38], whereas other studies have shown higher physical activity among inpatients [35]. Two, in the studies by Petzold et al. [39] and Chapman et al. [37], the patients were recruited by flyers and doctors, resulting perhaps in higher rates of physical activity due to self-motivation and potentially excluding patients with no interest in physical activity. In our analysis, all patients admitted to the wards were included, showing a more realistic view by decreasing selection bias.

Another outpatient study by Helgadóttir et al. [40] found an even higher physical activity level, with an average of 41.6 min of moderate to intense physical activity per day. This study included patients with anxiety disorder and mood (affective) diagnoses. Our study showed that these patient groups had the highest physical activity levels.

Compared to the abovementioned studies, other studies found lower physical activity levels when measured by an accelerometer [41–43]. However, these studies still measured higher levels of physical activity than our study. In one of these studies—using an accelerometer—Jerome et al. took a closer look at the duration of each bout of moderate to vigorous physical activity performed by patients. They distinguished between bouts of 10 min or higher and bouts of any duration (including less than 10 min). When counting bouts of any duration, they found an average of 120 min per week of moderate to vigorous physical activity. However, when only counting bouts of activity with a duration of 10 min or higher, they found an average of 24.9 min per week of moderate to vigorous physical activity performed by patients [41]. This is important when considering the results of this study. When asking patients to recall their physical activity, they are most likely to remember only moderate to vigorous activities that exceed 10 min at a time. This phenomenon may explain the lower physical activity levels found in our study.

Another factor to consider when assessing the results of this study is the individual rather than the overall increase in physical activity. An increase of 19.04 min of moderate to vigorous physical activity per week may seem rather small, however, it is important to consider that only about one-third of the patients engaged in any physical activity upon discharge. This means that these patients engaging in any physical activity actually increased their physical activity levels by roughly 60 min per week. In general, however, clinicians should be aware that the WHO has stated that even small increases in moderate to vigorous physical activity are beneficial to patient health. Therefore, every increase in physical activity is a win [31].

Only 9.5% of patients in our study complied with the WHO recommendation upon admission and 14.6% upon discharge. These numbers are meagre, considering the positive effects physical activity can provide. Moreover, even though the number of patients being physically inactive decreased from 76.2 to 61.9%, showing potential improvement through inpatient treatment, almost two-thirds of patients remain physically inactive. Thus, more systematic interventions are needed to increase physical activity throughout inpatient treatment.

Coleman et al. analysed the exercise as a vital sign (EVS) data of patients in primary care. They found that 36% of patients were inactive, 33% were insufficiently active, and 31% were sufficiently physically active [27]. These findings show a higher percentage of patients engaging insufficiently and sufficiently in physical activity compared to our findings. This difference can be explained by Kruisdijk et al. They showed that healthy individuals, as found in the primary care setting, show higher physical activity compared to patients with mental disorders- the cohort of patients in our study [20].

### Psychiatric diagnoses

Patients with neurotic, anxiety, stress-related, somatoform disorder or mood (affective) disorder diagnoses showed a higher physical activity level than patients with other mental disorders. Our finding of low physical activity in patients with schizophrenic, schizotypal, or delusional disorders supports the findings by Vancampfort et al. that patients with schizophrenia show lower physical activity compared to patients with other psychiatric diagnoses [35].

Furthermore, throughout inpatient treatment, patients with a schizophrenic, schizotypal, or delusional disorder diagnosis continued to show a low physical activity level. In contrast, patients with neurotic, anxiety, stress-related, or somatoform disorder diagnoses or a mood (affective) disorder diagnosis further increased the number of minutes spent performing moderate to vigorous physical activity per week. One possible explanation could be that patients with schizophrenia participate in sports therapy significantly less [36] compared to patients with other mental disorders. This might explain the little to no increase in physical activity during inpatient treatment. Another explanation may be an inadequate offer of physical activity interventions for patients with schizophrenic disorders offered during inpatient treatment. The needs of patients with schizophrenic disorders may differ from others and should therefore be explicitly catered to this patient group. Further reasons might be limitations due to higher disease severity and comorbidities associated with schizophrenia or side effects of antipsychotic medication. These factors reinforce the importance of implementing a more specific approach to increase the physical activity of patients with schizophrenia.

### Wards

The open ward showed the highest physical activity upon admission, followed by the day-clinic ward. The optionally closed wards showed the lowest physical activity. During inpatient treatment, patients on the day-clinic ward showed a significantly higher increase in physical activity compared to the open and optionally closed wards. On one optionally closed ward, we saw only a slight increase in physical activity. On the other optionally closed ward, we saw a slight decrease in physical activity. Patients in the open ward further increased their physical activity.

A reason for patients’ higher physical activity levels on the open and day-clinic ward may be the lower severity of their mental illness, as patients with severe mental illness tend to be less active. Another explanation for the increased physical activity among patients on the day-clinic ward may be the daily journey to the clinic and back, as some patients might have used bicycles. The free time outside the clinic could have also allowed patients to participate in further physical activities.

To explain the difference between the two optionally closed wards, a closer look at the patient distribution is essential. Optionally closed ward 1 (OCW1) is primarily for patients with mood (affective) disorders, whereas optionally closed ward 2 (OCW2) is primarily for patients with schizophrenic, schizotypal, and delusional disorders. As we showed that patients with schizophrenic, schizotypal, and delusional disorders show lower physical activity than patients with mood (affective) disorders, the difference between these two wards can be explained.

The higher physical activity among patients on the open and day-clinic wards still raises the question of how to increase physical activity on optionally closed wards. Further studies on specific differences between wards and these patients would be needed to address this.

### Exploratory analysis

We found that education, housing situation, employment status, legal guardianship status, residence status, month of admission, year of admission, alcohol intake, nicotine use, drug use, depression severity and duration of treatment were associated with the level of physical activity. Age, gender, BMI and comorbidities showed no significant association with the level of physical activity.

The exploratory analysis indicates that many factors might influence physical activity in patients with mental disorders. The benefit gained by identifying and verifying these factors could be enormous. Upon admission, patients with different needs to increase physical activity could be identified and selected for specific interventions such as individual or group training.

Many factors we identified might seem obvious (such as education and status of employment). Other factors, like the month of admission, seem less obvious. This, however, may indicate the need for new approaches in specific months of the year—especially in the winter. A larger cohort would be needed to verify the factors that we found in this study. This need reinforces the importance of implementing physical activity as a vital sign because only through data can we create an evidence-based approach to meeting the needs of all patients with psychiatric disorders.

### Implementing physical activity as a vital sign

Implementing physical activity as a vital sign in everyday clinical practice is an ongoing procedure. Constant meetings with clinicians are needed to explain the importance of implementing physical activity as a vital sign to ensure ongoing documentation. One reason for the difficulty of implementing this tool might be rooted in the lack of education regarding physical activity in medical schools [44, 45]. Physicians need to be aware of the importance of physical activity and engage in more physical activity themselves. There is a strong positive correlation between the physical activity habits and good lifestyle choices of medical students and doctors and their attitude towards physical activity counselling [46, 47]. Patients are also more likely to participate in preventive practices when physicians do so [47]. As we showed in the results section, documentation of physical activity has not been achieved for every patient, and the documentation rate fluctuated in all wards. Hence, optimising the documentation of physical activity is crucial. As Electronic Health Records and reminder systems led to more frequent energy balance counselling in primary care [48], implementing these should be considered to obtain a higher rate of documenting physical activity levels.

### Strengths and limitations

One strength of this paper is the selection of patients. As we included all patients on all psychiatric wards, the study cohort is a heterogeneous group. In addition, patients were not actively recruited, thereby minimising selection bias.

However, it is possible that a slight selection bias occurred. Some severely ill patients were in no state to answer questions concerning physical activity and were, therefore, not included in this analysis. Documenting the diagnosis of patients with missing physical activity data could help address this aspect in future studies. Important to note is that during the implementation phase of documenting physical activity levels (during which this study took place), physician adherence to the documentation of physical activity levels could influence data.

One limitation of this analysis is the small number of patients, with only 328 patients meeting all of our inclusion criteria. Therefore, results concerning subgroups with smaller patient numbers should be evaluated carefully. In addition, the factors we identified that might influence the level of physical activity should be considered with caution as more patients are needed to verify these factors. In general, due to its design and the limited statistical analyses, our study only provides explorative information which nevertheless lays an important basis for future studies.

Using a self-report physical activity tool might be another limitation, as patients might state higher or lower physical activity levels than performed. Indeed, previous studies showed that physical activity levels measured with an accelerometer are lower than using a self-report questionnaire [17, 49]. However, according to one study by Tucker et al., this is not true when using exercise as a vital sign (EVS) or physical activity as a vital sign (PAVS) [49]. Physical activity measured with the EVS is even lower than when measured with an accelerometer [50]. Even though the EVS does not fully correlate with the accelerometer-measured physical activity, it showed a high specificity (74–89%) [28] and an accurate negative predictive value [50], making it a fitting tool to identify patients who are not meeting the physical activity guidelines.

Unfortunately, the translation of the EVS into the German language has not yet been validated. This would be an important aspect to investigate in future studies.

Important for future studies on this topic might be to document the clinic’s sports program throughout the year for each ward. This information could help to understand changes in physical activity levels associated with the clinic’s sports program, thereby showing ways of proactively increasing physical activity levels and enabling comparison between clinics.

## Outlook and perspective

An evidence-based foundation is crucial to increase physical activity, thereby decreasing the incidence and prevalence of mental disorders [32, 33]. To achieve this, regular assessment of physical activity levels by medical professionals in primary and secondary health care is needed [33]. By implementing physical activity in the standard documentation of patients in the department of psychiatry and psychotherapy at the Campus Charité Mitte, we want to carve the path of regular data collection on physical activity levels on a broad scale. Doing so will allow us to continue collecting non-biased data that could help the development of evidence-based approaches for subgroups of psychiatric patients. Furthermore, by sharing our experience and the tools we have used, we hope to help other health centres implement similar frameworks.

EVS is a tremendous tool for this purpose as it is a validated and time-efficient tool to evaluate the physical activity levels of an extensive range of patient groups [27, 51]. Not only does a more frequent assessment lead to an increase in positive lifestyle changes [52], but the time-efficient manner of this tool enables frequent follow-ups, consolidating this increase with each patient visit [53].

However, physical activity assessment is only the first step in creating a foundation for evidence-based approaches. Patients not meeting the recommendation of 150 min of moderate to intense physical activity should be referred to fitness or exercise professionals with specific approaches that target individual patient groups.

In the primary health care setting, multiple studies showed a positive effect of physical activity counselling and interventions such as exercise referral schemes [53–55]. Interventions with a multiple-behavioural-change approach and a face-to-face delivery of standardised interventions were found to be more effective in increasing physical activity [53]. A multifaceted approach is needed to implement this in psychiatry, involving government, communities and health care professionals. At the forefront of patient care, a closer link between psychiatrists, physicians and fitness professionals will enable interdisciplinary encouragement of physical activity [31, 33, 56].

## Conclusion

This study showed that many patients increased their physical activity levels during inpatient treatment. This increase highlights the window of opportunity that inpatient treatment presents regarding the potential intervention of increasing physical activity. On the other hand, not all patients increased their physical activity. Most notably, we saw differences between different groups of mental disorders. Other potential factors that may influence physical activity were identified as well. Further studies will be needed to confirm these.

Our findings reinforce the importance of gathering data on physical activity in psychiatric patients. Only through this will we find evidence-based approaches suitable for patients with different mental disorders. Assessing physical activity by asking two simple questions gives us this opportunity.

By increasing their physical activity, patients can actively play a part in managing their disease. Physicians can encourage this, thereby empowering patients and increasing adherence to recommended lifestyle choices. Evidence-based data is necessary to establish structures that will allow physicians and patients to develop beneficial behaviour patterns regarding physical activity. Patients can make informed choices by physicians openly discussing the benefits of physical activity, adjusted explicitly to psychiatric diagnoses. A statement made by Robert Sallis can summarise the importance of this conversation: “At that point, patients can at least make an informed decision about how active they choose to be” [56].

## Supplementary Information

Below is the link to the electronic supplementary material.

Supplementary file1 (DOCX 49 KB)

## Data Availability

Data are available from the corresponding author upon reasonable request.
