# Supplementary material for: Tracking changes in physical activity during inpatient treatment in a psychiatric clinic in Germany by asking two simple questions
Source: Eur Arch Psychiatry Clin Neurosci. 2023 Feb 11;273(4):983–94. doi: 10.1007/s00406-023-01565-2 (PMC9918842; doi:10.1007/s00406-023-01565-2)
Supplement: Supplementary file 1 — Supplementary file1 (DOCX 49 KB) [file 406_2023_1565_MOESM1_ESM.docx]

Tracking changes in physical activity during inpatient treatment in a psychiatric clinic in Germany by asking two simple questions

Jannik Roempler^a^, Moritz Bruno Petzold^a^, Antonia Bendau^a,b^, Jens Plag^c^, Andreas Ströhle^a*^

^a^ Charité – Universitätsmedizin Berlin, corporate member of Freie Universität Berlin and Humboldt Universität zu Berlin, Department of Psychiatry and Neurosciences, CCM, Charitéplatz 1, 10117, Berlin, Germany

^b^ HMU Health and Medical University Potsdam, Germany

^c^ Oberberg Fachklinik Potsdam, Potsdam, Germany

ORCID: Jannik Roempler: 0000-0003-2271-0567

ORCID: Moritz Bruno Petzold: 0000-0002-7801-1434

ORCID: Antonia Bendau: 0000-0002-3789-6205

ORCID: Andreas Ströhle: 0000-0003-0935-3702

To be submitted to: European Archives of Psychiatry and Clinical Neuroscience

*Corresponding author:

Andreas Ströhle

Charité – Universitätsmedizin Berlin, corporate member of Freie Universität Berlin and Humboldt Universität zu Berlin, Department of Psychiatry and Neurosciences,

Charitéplatz 1, 10117 Berlin, Germany

Tel.: +49 30 450 517034

Fax: +49 30 450 517934

E-Mail: andreas.stroehle@charite.de

Supplements - Implementation of physical activity in the standard documentation of a psychiatric clinic in Germany

**Table S1**

*Physical activity in different mental disorders*

| **F-Diagnosis** | **PA upon admission (min/week)** | | | | |
| --- | --- | --- | --- | --- | --- |
|  | Mean (SD) | Median | Minimum | Maximum | *p* |
|  |  |  |  |  | .009** |
| **F0 (n=6)** | 23.33 (36.70) | 0 | 0 | 80 |  |
| **F1 (n=63)** | 35.24 (88.00) | 0 | 0 | 420 |  |
| **F2 (n=86)** | 10.12 (33.00) | 0 | 0 | 180 |  |
| **F3 (n=140)** | 52.14 (115.90) | 0 | 0 | 900 |  |
| **F4 (n=18)** | 111.11 (170.15) | 0 | 0 | 420 |  |
| **F6 (n=11)** | 43.64 (126.12) | 0 | 0 | 420 |  |
| **F9 (n=2)** | 0 (0) | 0 | 0 | 0 |  |
| **F-Diagnosis** | **PA upon discharge (min/week)** | | | | |
|  | Mean (SD) | Median | Minimum | Maximum | *p* |
|  |  |  |  |  | <.001*** |
| **F0 (n=6)** | 33.33 (57.50) | 0 | 0 | 140 |  |
| **F1 (n=63)** | 39.21 (90.52) | 0 | 0 | 420 |  |
| **F2 (n=86)** | 16.98 (39.42) | 0 | 0 | 150 |  |
| **F3 (n=140)** | 87.68 (123.72) | 60 | 0 | 900 |  |
| **F4 (n=18)** | 130.00 (157.11) | 60 | 0 | 420 |  |
| **F6 (n=11)** | 46.36 (126.83) | 0 | 0 | 420 |  |
| **F9 (n=2)** | 0 (0) | 0 | 0 | 0 |  |
| **F-Diagnosis** | **Increase in PA (min/week)** | | | | |
|  | Mean (SD) | Median | Minimum | Maximum | *p* |
|  |  |  |  |  | <.001*** |
| **F0 (n=6)** | 10.00 (24.50) | 0 | 0 | 60 |  |
| **F1 (n=63)** | 3.97 (37.91) | 0 | -120 | 190 |  |
| **F2 (n=86)** | 6.86 (32.12) | 0 | -90 | 150 |  |
| **F3 (n=140)** | 35.54 (77.73) | 0 | -300 | 375 |  |
| **F4 (n=18)** | 18.89 (48.68) | 0 | -60 | 150 |  |
| **F6 (n=11)** | 2.73 (9.05) | 0 | 0 | 30 |  |
| **F9 (n=2)** | 0 (0) | 0 | 0 | 0 |  |

*Note.* The moderate to vigorous physical activity (PA) for patients with different mental disorders is shown upon admission and discharge. Additionally, the increase in PA is shown in the lower third of the table. In the table, the means, medians, minimums, maximums, and standard deviations (SD) can be found. F0=organic, including symptomatic, mental disorders; F1= mental and behavioral disorders due to psychoactive substance abuse; F2= schizophrenia, schizotypal, and delusional disorders; F3= mood disorders, depression, and bipolar disorders; F4= neurotic, anxiety, stress-related, and somatoform disorders; F6= disorders of adult personality and behaviors; F9= behavioral and emotional disorders with onset usually occurring in childhood and adolescence. Significance of the differences between mental disorders was calculated by Kruskal Wallis Tests.

**Table S2**

*Physical activity on different wards*

| **Ward** | **PA upon admission (min/week)** | | | | |
| --- | --- | --- | --- | --- | --- |
|  | Mean (SD) | Median | Minimum | Maximum | *p* |
|  |  |  |  |  | <.001*** |
| **OCW1 (n=90)** | 39.61 (91.43) | 0 | 0 | 420 |  |
| **OW (n=18)** | 147.78 (165.09) | 80 | 0 | 420 |  |
| **DCW (n=96)** | 47.66 (121.51) | 0 | 0 | 900 |  |
| **OCW2 (n=124)** | 17.82 (57.74) | 0 | 0 | 300 |  |
| **Ward** | **PA upon discharge (min/week)** | | | | |
|  | Mean (SD) | Median | Minimum | Maximum | *p* |
|  |  |  |  |  | <.001*** |
| **OCW1 (n=90)** | 51.39 (86.16) | 0 | 0 | 420 |  |
| **OW (n=18)** | 164.44 (176.84) | 80 | 0 | 420 |  |
| **DCW (n=96)** | 99.32 (130.32) | 60 | 0 | 900 |  |
| **OCW2 (n=124)** | 17.22 (55.82) | 0 | 0 | 300 |  |
| **Ward** | **Increase in PA (min/week)** | | | | |
|  | Mean (SD) | Median | Minimum | Maximum | *p* |
|  |  |  |  |  | <.001*** |
| **OCW1 (n=90)** | 11.78 (52.30) | 0 | -300 | 140 |  |
| **OW (n=18)** | 16.67 (86.84) | 0 | -60 | 360 |  |
| **DCW (n=96)** | 51.67 (74.80) | 17.5 | 0 | 375 |  |
| **OCW2 (n=124)** | -0.60 (24.38) | 0 | -120 | 150 |  |

*Note.* In the table the moderate to vigorous physical activity (PA) on the optionally closed ward 1 (OCW1), open ward (OW), day clinic ward (DCW) and optionally closed ward 2 (OCW2) is shown upon admission and discharge. Additionally, the increase in PA is shown in the lower third of the table. In the table, the means, medians, minimums, maximums, and standard deviations (Std. Dev.) can be found. Significance of the differences between mental disorders was calculated by Kruskal Wallis Tests.

**Table S3**

*Factors that might be associated with the level of physical activity*

| **Variable**  Groups (n) | **PA upon admission (min/week)** | | | **PA upon discharge (min/week)** | | | **Increase in PA (min/week)** | | |
| --- | --- | --- | --- | --- | --- | --- | --- | --- | --- |
|  | Mean (SD) | Med. | *p* | Mean | Med. | *p* | Mean | Med. | *p* |
| **Gender ^a)^** |  |  | .769 |  |  | .899 |  |  | .326 |
| Male (176) | 38.69 (90.47) | 0 |  | 56.88 (98.00) | 0 |  | 18.18 (59.57) | 0 |  |
| Female (152) | 40.79 (111.07) | 0 |  | 60.82 (117.52) | 0 |  | 20.0 (58.10) | 0 |  |
| **BMI ^b)^** |  |  | .094 |  |  | .062 |  |  | .878 |
| Underweight (3) | 0 (0) | 0 |  | 20.00 (34.64) | 0 |  | 20.00 (34.64) | 0 |  |
| Normal weight (42) | 97.74 (182.87) | 0 |  | 144.64 (175.62) | 105 |  | 46.90 (76.53) | 0 |  |
| Overweight (23) | 23.04 (48.38) | 0 |  | 67.61 (87.89) | 60 |  | 44.57 (84.40) | 0 |  |
| Adipose (17) | 26.76 (51.69) | 0 |  | 77.65 (76.61) | 60 |  | 50.88 (69.33) | 15 |  |
| **Education ^b)^** |  |  | .002** |  |  | <.001*** |  |  | .033* |
| No qualification (18) | 0 (0) | 0 |  | 6.67 (28.28) | 0 |  | 6.67 (28.28) | 0 |  |
| Secondary modern school qualification (8) | 7.50 (21.21) | 0 |  | 33.75 (54.23) | 0 |  | 26.25 (54.23) | 0 |  |
| High-school diploma (17) | 22.94 (49.21) | 0 |  | 14.12 (32.03) | 0 |  | -8.82 (40.76) | 0 |  |
| Higher School Certificate (25) | 27.20 (67.05) | 0 |  | 51.80 (79.41) | 0 |  | 24.60 (61.68) | 0 |  |
| Completed vocational training (84) | 32.44 (93.84) | 0 |  | 52.14 (93.59) | 0 |  | 19.70 (56.50) | 0 |  |
| With a degree (103) | 64.71 (129.90) | 0 |  | 97.77 (139.26) | 60 |  | 33.06 (78.05) | 0 |  |
| **Housing situation ^b)^** |  |  | <.001*** |  |  | <.001*** |  |  | .001** |
| Without a fixed abode (55) | 5.45 (28.92) | 0 |  | 7.91 (28.70) | 0 |  | 2.45 (24.79) | 0 |  |
| Therapeutical housing (16) | 33.75 (105.00) | 0 |  | 40.63 (105.86) | 0 |  | 6.88 (20.89) | 0 |  |
| Private (227) | 50.22 (112.98) | 0 |  | 75.20 (119.23) | 0 |  | 24.98 (67.19) | 0 |  |
| **Status of employment ^b)^** |  |  | .003** |  |  | <.001*** |  |  | .028* |
| Yes (76) | 75.26 (145.82) | 0 |  | 105.00 (146.53) | 0 |  | 29.74 (66.62) | 0 |  |
| No (150) | 26.97 (73.98) | 0 |  | 44.90 (86.46) | 0 |  | 17.93 (52.64) | 0 |  |
| In education (31) | 32.42 (86.96) | 0 |  | 58.71 (97.22) | 0 |  | 26.29 (57.50) | 0 |  |
| Retired (53) | 36.60 (97.41) | 0 |  | 42.83 (96.74) | 0 |  | 6.23 (70.20) | 0 |  |
| **Legal Guardianship ^a)^** |  |  | .025* |  |  | .001** |  |  | .032* |
| Yes (71) | 19.30 (63.88) | 0 |  | 32.54 (82.39) | 0 |  | 13.24 (60.14) | 0 |  |
| No (256) | 45.47 (107.93) | 0 |  | 66.19 (112.46) | 0 |  | 20.72 (58.56) | 0 |  |
| **Residence status ^a)^** |  |  | .579 |  |  | .066 |  |  | .208 |
| Voluntary (305) | 40.93 (103.24) | 0 |  | 61.10 (109.86) | 0 |  | 20.16 (60.09) | 0 |  |
| Nonvoluntary (21) | 21.43 (47.57) | 0 |  | 22.86 (54.42) | 0 |  | 1.43 (34.83) | 0 |  |
| **Month of admission ^b)^** |  |  | .222 |  |  | .001** |  |  | .004** |
| January (40) | 26.00 (72.92) | 0 |  | 24.88 (60.61) | 0 |  | -1.13 (31.20) | 0 |  |
| February (24) | 42.71 (102.16) | 0 |  | 63.33 (106.31) | 0 |  | 20.63 (33.63) | 0 |  |
| March (28) | 18.75 (48.41) | 0 |  | 21.61 (41.03) | 0 |  | 2.86 (32.42) | 0 |  |
| April (16) | 18.75 (60.87) | 0 |  | 15.00 (60.00) | 0 |  | -3.75 (15.00) | 0 |  |
| May (22) | 71.82 (191.50) | 0 |  | 78.64 (194.60) | 0 |  | 6.82 (34.56) | 0 |  |
| June (27) | 21.67 (53.33) | 0 |  | 46.11 (76.25) | 0 |  | 24.44 (42.66) | 0 |  |
| July (31) | 77.10 (145.90) | 0 |  | 112.74 (161.29) | 50 |  | 35.65 (95.66) | 0 |  |
| August (27) | 70.74 (126.52) | 0 |  | 113.89 (118.67) | 100 |  | 43.15 (65.91) | 0 |  |
| September (32) | 39.22 (87.36) | 0 |  | 68.59 (78.63) | 0 |  | 29.38 (85.92) | 0 |  |
| October (14) | 5.00 (12.86) | 0 |  | 55.71 (85.55) | 0 |  | 50.71 (79.85) | 0 |  |
| November (36) | 18.33 (47.72) | 0 |  | 39.44 (77.05) | 0 |  | 21.11 (67.14) | 0 |  |
| December (31) | 53.87 (107.17) | 0 |  | 63.06 (109.29) | 0 |  | 9.19 (28.93) | 0 |  |
| **Year of admission ^b)^** |  |  | .181 |  |  | .001** |  |  | .002** |
| 2019 (89) | 51.80 (105.55) | 0 |  | 96.24 (125.34) | 30 |  | 44.44 (85.38) | 0 |  |
| 2020 (232) | 34.31 (97.22) | 0 |  | 44.18 (95.98) | 0 |  | 9.87 (41.77) | 0 |  |
| 2021 (7) | 62.86 (134.38) | 0 |  | 62.86 (116.86) | 0 |  | 0.00 (34.64) | 0 |  |
| **Alcohol use current ^b)^** |  |  | .024* |  |  | .063 |  |  | .282 |
| No consumption (184) | 34.89 (89.83) | 0 |  | 60.54 (103.23) | 0 |  | 25.65 (69.57) | 0 |  |
| Irregular consumption (43) | 89.42 (165.08) | 0 |  | 96.16 (161.27) | 60 |  | 6.74 (42.62) | 0 |  |
| Regular consumption (35) | 18.86 (41.71) | 0 |  | 36.00 (59.52) | 0 |  | 17.14 (41.84) | 0 |  |
| Consumption disorder (64) | 32.58 (87.57) | 0 |  | 42.50 (89.22) | 0 |  | 9.92 (37.58) | 0 |  |
| **Alcohol use history ^b)^** |  |  | .028* |  |  | .053 |  |  | .715 |
| No consumption (160) | 34.91 (92.90) | 0 |  | 57.25 (104.52) | 0 |  | 22.34 (70.07) | 0 |  |
| Irregular consumption (40) | 93.88 (170.05) | 0 |  | 103.38 (165.05) | 60 |  | 9.50 (41.31) | 0 |  |
| Regular consumption (43) | 22.33 (48.05) | 0 |  | 48.14 (69.36) | 0 |  | 25.81 (55.69) | 0 |  |
| Consumption disorder (83) | 32.65 (82.45) | 0 |  | 46.87 (89.85) | 0 |  | 14.22 (41.85) | 0 |  |
| **Nicotine use current ^b)^** |  |  | .250 |  |  | .037* |  |  | .044* |
| No consumption (198) | 39.22 (90.20) | 0 |  | 63.11 (99.60) | 0 |  | 23.89 (67.92) | 0 |  |
| Irregular consumption (10) | 75.00 (139.46) | 0 |  | 76.00 (140.10) | 0 |  | 1.00 (3.16) | 0 |  |
| Regular consumption (112) | 39.33 (115.93) | 0 |  | 49.55 (119.62) | 0 |  | 10.00 (40.11) | 0 |  |
| **Nicotine use history ^b)^** |  |  | .253 |  |  | .099 |  |  | .048 |
| No consumption (188) | 38.22 (90.85) | 0 |  | 62.63 (100.97) | 0 |  | 24.41 (68.74) | 0 |  |
| Irregular consumption (11) | 79.09 (133.00) | 0 |  | 80.00 (133.57) | 0 |  | 0.91 (3.02) | 0 |  |
| Regular consumption (121) | 40.21 (113.44) | 0 |  | 50.83 (116.73) | 0 |  | 10.62 (41.11) | 0 |  |
| **Drug use current ^b)^** |  |  | .224 |  |  | .037* |  |  | .130 |
| No consumption (240) | 41.85 (93.51) | 0 |  | 64.19 (102.47) | 0 |  | 22.33 (64.32) | 0 |  |
| Irregular consumption (19) | 79.74 (222.38) | 0 |  | 90.79 (221.16) | 0 |  | 11.05 (27.31) | 0 |  |
| Regular consumption (12) | 10.00 (34.64) | 0 |  | 27.50 (50.30) | 0 |  | 17.50 (41.37) | 0 |  |
| Drug abuse (56) | 23.75 (66.68) | 0 |  | 30.98 (69.79) | 0 |  | 7.23 (41.95) | 0 |  |
| **Drug use history ^b)^** |  |  | .249 |  |  | .290 |  |  | .209 |
| No consumption (173) | 40.20 (89.57) | 0 |  | 57.02 (96.35) | 0 |  | 16.82 (56.25) | 0 |  |
| Irregular consumption (48) | 68.23 (166.79) | 0 |  | 92.71 (169.95) | 0 |  | 24.48 (60.09) | 0 |  |
| Regular consumption (40) | 18.75 (54.22) | 0 |  | 51.75 (85.69) | 0 |  | 33.00 (82.06) | 0 |  |
| Drug abuse (66) | 30.76 (81.34) | 0 |  | 42.58 (84.78) | 0 |  | 11.82 (46.30) | 0 |  |
| **Depression ^b)^** |  |  | .141 |  |  | <.001*** |  |  | <.001*** |
| No depression (184) | 35.43 (105.65) | 0 |  | 40.65 (106.09) | 0 |  | 5.22 (31.33) | 0 |  |
| Bipolar disorder depression (19) | 64.21 (130.91) | 0 |  | 78.95 (109.23) | 50 |  | 14.74 (105.01) | 0 |  |
| Mild depression (6) | 45.00 (70.36) | 0 |  | 61.67 (76.00) | 30 |  | 16.67 (24.22) | 5 |  |
| Moderate depression (69) | 31.88 (58.12) | 0 |  | 70.36 (78.79) | 60 |  | 38.48 (68.12) | 0 |  |
| Severe depression (49) | 57.14 (116.75) | 0 |  | 103.06 (134.20) | 60 |  | 45.92 (83.81) | 0 |  |
| **Age categories ^b)^** |  |  | .198 |  |  | .646 |  |  | .892 |
| 18-30 (67) | 49.10 (100.75) | 0 |  | 67.16 (103.99) | 0 |  | 18.06 (51.58) | 0 |  |
| 31-40 (82) | 38.66 (115.13) | 0 |  | 57.56 (121.44) | 0 |  | 18.90 (60.68) | 0 |  |
| 41-50 (78) | 22.18 (69.56) | 0 |  | 43.53 (81.20) | 0 |  | 21.35 (47.87) | 0 |  |
| 51-64 (74) | 50.27 (115.54) | 0 |  | 67.57 (119.79) | 0 |  | 17.30 (67.31) | 0 |  |
| >64 (27) | 40.74 (80.09) | 0 |  | 60.74 (102.77) | 0 |  | 20.00 (75.80) | 0 |  |
| **Duration of treatment ^c)^** |  |  | <0.01** |  |  | <0.01** |  |  | <0.01** |

*Note.* This table shows the moderate to vigorous physical activity (PA) in minutes/week in relation to variables. a) Significance of the differences between the two groups was calculated by Mann-Whitney U Tests. b) Significance of the differences between the three or more groups was calculated by Kruskal Wallis Tests. c) Significance of the association with physical activity was calculated by Spearman’s Rank Correlation.
